# Supplementary material for: Cloning and Characterization of Aedes aegypti Trypsin Modulating Oostatic Factor (TMOF) Gut Receptor
Source: Biomolecules. 2021 Jun 23;11(7):934. doi: 10.3390/biom11070934 (PMC8301768; doi:10.3390/biom11070934)
Supplement: Supplementary file 1 [file biomolecules-11-00934-s001.zip › biomolecules-1271958-supplementary.pdf]

Supplementary material

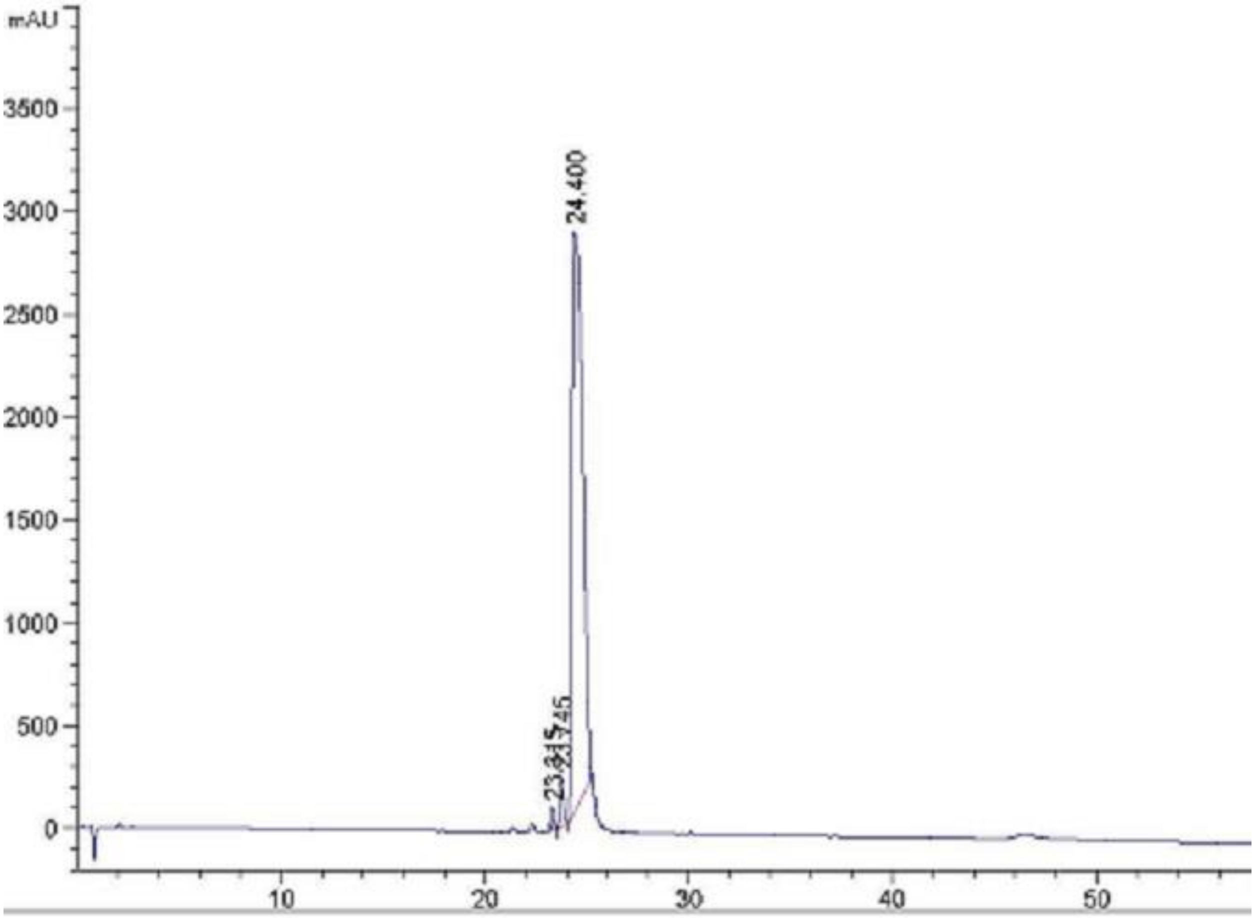

S1. Purification of TMOF-FITC by HPLC

```
Spectrum Deconvolution
Data File D:\Data\TD20190829A\td 2019-08-29 10-32-00\012-P1-A2-Brovosky-Pure.D
Sample Name: Brovosky-Pure
```

Deconvolution of Spectrum # 1 @ 24.489 min

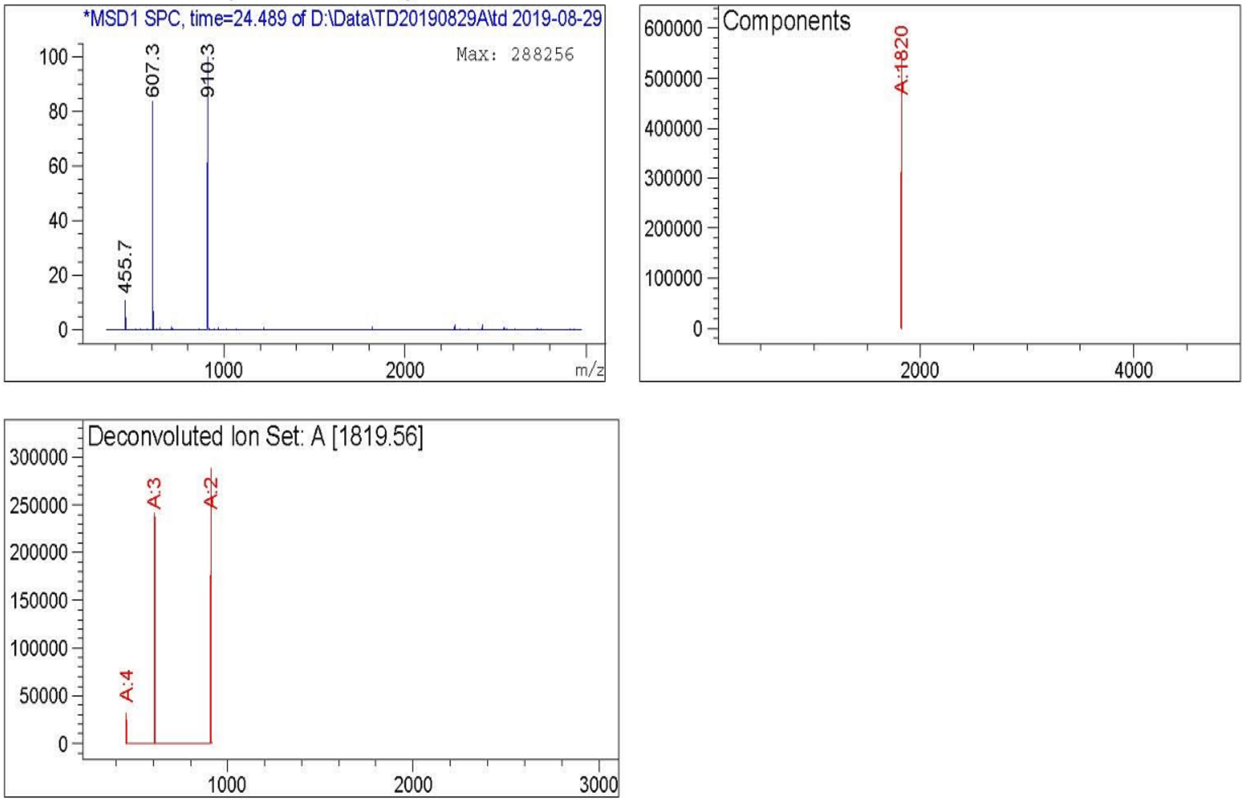

Component	Molecular Weight	Absolute Abundance	Relative Abundance
A	1819.56	550224	100.00

*** End of Report ***

S2. Mass spectrometry of purified TMOF-FITC after HPLC purification, note the distinct expected ions at 455.7, 607.3 and 910.3.

```
901IMLESWYTESSGLKEKQSLESAIKLAVEAISNIRTVASLGQEPYVLERYYKEIAKVD
ACKKKSRLRGVVFALGQIMPFMGYGLALFYGGKLVSEAELEYKDVIKVSEALIFGAWMLG
ALAYAPNVNSAMLSAGRLMKLLDRTPKMHNPSSSYLSTFENHEGNIKFTDVEFRYPTRPT
PILQGLNLDIKKGNTVALVGPSGCGKSTCIQLLLRYYDPDNGKVDIDGITTTDFQLGRIR
QMGLVSQEPVLFDRTIAENIAYGDNTREISMPEIIEASKMANIHEFIVNLPKGYDTSLGT
GAQLSGGQKQRIAIARALVRNPRILLLDEATSALDNQSEKIVQNALDHARKGRTCIIIAH
LTTIQNADLICVIQSGVVVECGTHDELMAQNKIYAKLYAMQQVA1304
```

S3. *Aea*ABC-TMOF receptor sequence of the 45 kDa SDS PAGE stained band (Figure 1B) corresponding to the C-terminal end of the *Aea*ABC-TMOF receptor sequence (Figure 3) is highlighted in yellow. The sequence highlighted in cyan corresponds to the α helical sequence involved in the binding of TMOF to its receptor (Figure 8 A,B). The sequence highlighted in red corresponds to MS/MS identified peptide. The ATP binding domain at the C-terminal of the receptor is underlined.

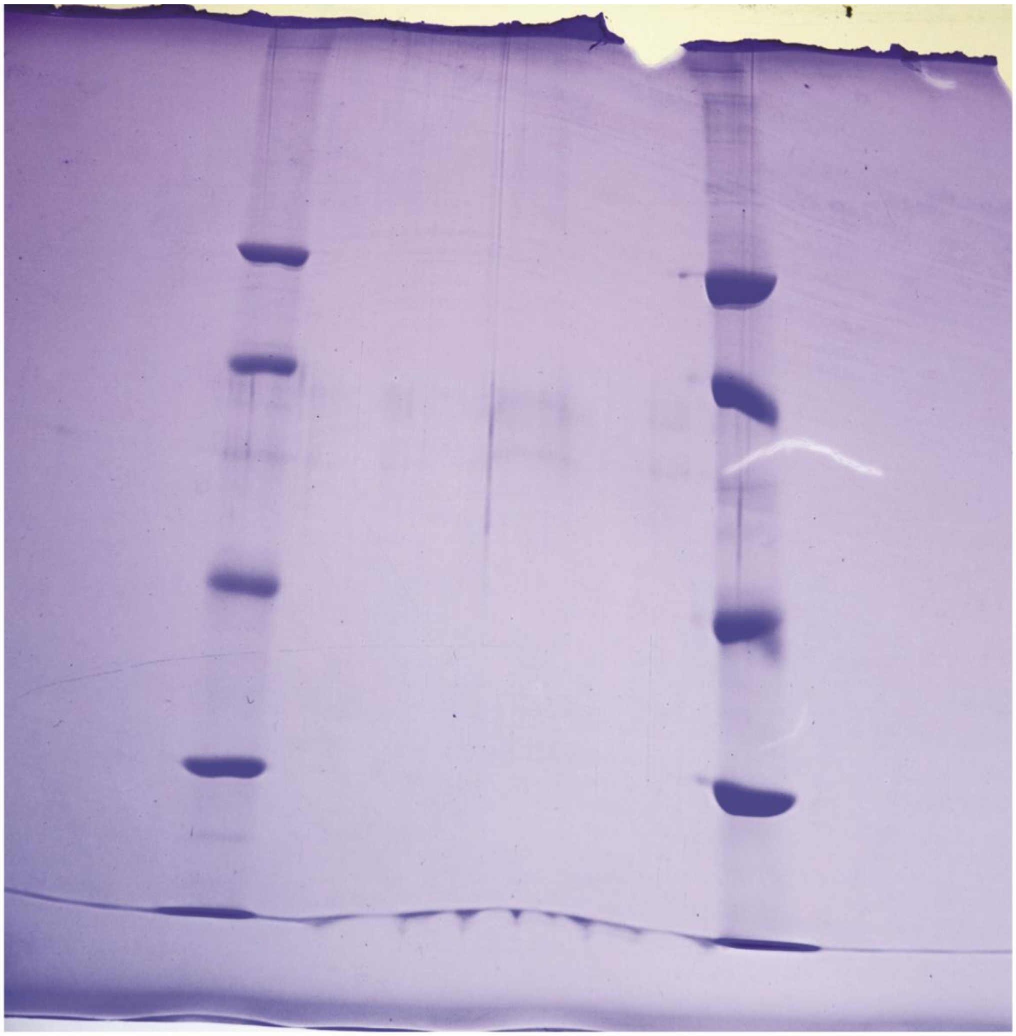

Original SDS PAGE that part was used for Figure 1B.
